# Supplementary material for: A Small Molecule Promoting Neural Differentiation Suppresses Cancer Stem Cells in Colorectal Cancer
Source: Biomedicines. 2022 Apr 6;10(4):859. doi: 10.3390/biomedicines10040859 (PMC9025482; doi:10.3390/biomedicines10040859)
Supplement: Supplementary file 1 [file biomedicines-10-00859-s001.zip › biomedicines-1645710-supplementary.pdf]

biomedicines

MDPI

Article

A Small Molecule Promoting Neural Differentiation Suppresses Cancer Stem Cells in Colorectal Cancer

Jung Kyu Choi [1,†], Ihn-Sil Kwak [2,†], Sae-Bom Yoon [3], Heeyeong Cho [3] and Byoung-San Moon [4,*]

1 Department of Biotechnology, College of Life and Applied Sciences, Yeungnam University, Gyeongsan, 38541, Korea; jungkyuc@ynu.ac.kr
2 Department of Ocean Integrated Science, Chonnam National University, Yeosu 59626, Korea; iskwak@jnu.ac.kr
3 Therapeutics and Biotechnology Division, Drug Discovery Platform Research Center, Korea Research Institute of Chemical Technology (KRICT), Daejeon, 34114, Korea; bomi9123@krict.re.kr (S.-B.Y.); hycho@krict.re.kr (H.C.)
4 Department of Biotechnology, Chonnam National University, Yeosu 59626, Korea
* Correspondence: bsmoon@jnu.ac.kr; Tel: 82-61-659-7307; Fax: 82-61-659-7309
† Authors are contributed equally to this work.

Citation: Choi, J.K.; Kwak, I.-S.; Yoon, S.-B.; Cho, H.; Moon, B.-S. A Small Molecule Promoting Neural Differentiation Suppresses Cancer Stem Cell in Colorectal Cancer. *Biomedicines* **2022**, *9*, 859. https://doi.org/10.3390/biomedicines10040859

Academic Editor: Luca Falzone

Received: 4 March 2022
Accepted: 5 April 2022
Published: 6 April 2022

Publisher's Note: MDPI stays neutral with regard to jurisdictional claims in published maps and institutional affiliations.

Copyright: © 2022 by the authors. Submitted for possible open access publication under the terms and conditions of the Creative Commons Attribution (CC BY) license (https://creativecommons.org/licenses/by/4.0/).

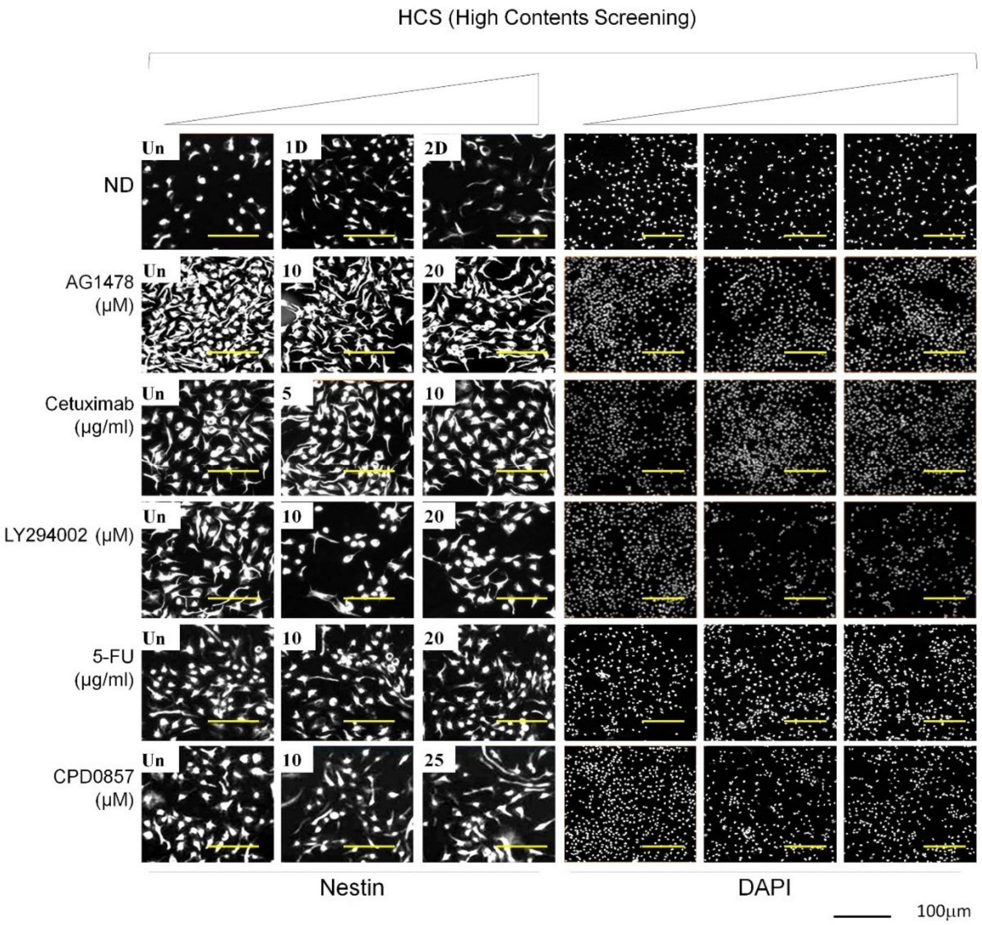

Figure S1. HCS analysis of drugs on proliferation and differentiation of the NPCs. HCS analysis of neurite outgrowth of NPCs. NPCs were grown in medium in the presence or absence of bFGF (20 ng/mL) and were treated with AG1478 (EGFR tyrosin kinase inhibitor, 10, 20 μM), Cetuximab (EGFR monoclonal antibody, 5, 10 μM), 5-FU (antineoplastic drug, 10, 20 ug/mL), LY294002 (PI3K/AKT inhibitor, 10, 20 μM), and CDP0857 (10, 25 μM) during 24 h. Cells were subjected to immunofluorescent labeling using antibody specific for Nestin. Nuclei were counterstained with DAPI. Images were obtained using a Cellomics HCS Array Scan. Scale bars, 100 μm. HCS, High-content screening.
